# Pulmonary sequestration in a septuagenarian: a rare congenital lung lesion diagnosed in adulthood

**DOI:** 10.1186/s13019-026-04098-6

**Published:** 2026-04-16

**Authors:** Saran Lal Ajai Mokan Dasan, Aura P Flores, Khaja Misbahuddin

**Affiliations:** Department of Internal Medicine, Bronxcare Health System, New York City, USA

**Keywords:** Pulmonary sequestration, Intralobar, Extralobar, Incidental lung mass

## Abstract

**Introduction:**

Pulmonary sequestration (PS) is a rare congenital pulmonary malformation characterized by non-functioning lung tissue that lacks normal communication with the tracheobronchial tree and receives systemic arterial blood supply. While it is often diagnosed in childhood due to recurrent infections, incidental detection in asymptomatic adults is uncommon and can pose a diagnostic challenge.

**Case details:**

We report the case of a 70-year-old female who was incidentally found to have a left lower lobe mass on routine imaging. Initial CT revealed a 3.2 cm triangular mass in the posteromedial aspect of the left lower lobe abutting the descending thoracic aorta. PET imaging showed the lesion to be non-FDG avid, and CT angiography demonstrated a feeding artery arising from the descending aorta, confirming the diagnosis of pulmonary sequestration. The patient was asymptomatic and declined surgical intervention. She is being managed conservatively with serial imaging, which has shown that the lesion remains stable over follow-up.

**Conclusion:**

Intralobar pulmonary sequestrants in older adults are rarely reported. Their close resemblance to malignancy in this population makes diagnosis challenging. Multimodal imaging, including PET and CTA, is crucial for accurate diagnosis. In asymptomatic patients without complications, conservative management with radiological surveillance may be a safe and appropriate approach.

## Introduction

Pulmonary sequestration (PS) is a rare congenital disease of the lower respiratory tract wherein a non-functioning bronchopulmonary mass, separate from the tracheobronchial tree, receives an anomalous feeding vessel from systemic circulation [1]. This anatomical peculiarity, coupled with absent bronchopulmonary clearance, leads to recurrent infections and, subsequently, when the need arises, renders surgical intervention more demanding [2]. Two forms exist, intralobar and extralobar. They differ in anatomy and clinical presentation. Pulmonary sequestration is estimated to occur in 1.1%-1.8% of patients undergoing lung resection. In adults, they are often incidentally detected on imaging performed for unrelated reasons. We present this case to highlight the unusual late-life diagnosis of asymptomatic pulmonary sequestration, which mimicked malignancy on imaging but was ultimately managed conservatively.

### Case details

**Fig. 1:**
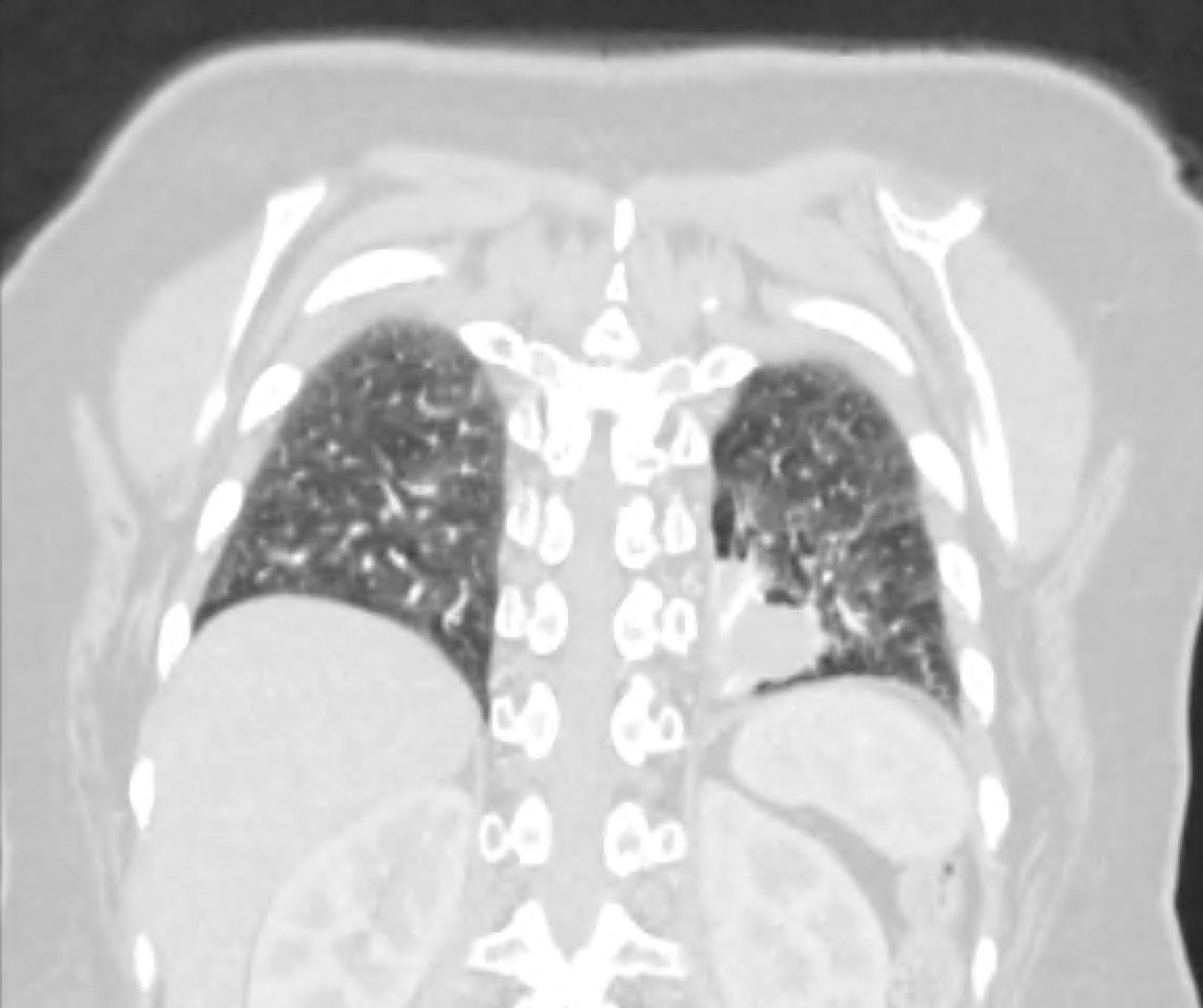
CT Chest coronal view showing left basal lung mass close to the spine

A 70-year-old female, a never-smoker with a history of hypertension, prediabetes, and thyroid nodules, was found to have a lung nodule in January 2023 on routine imaging. Review of symptoms was negative for respiratory/systemic symptoms such as dyspnea, chronic cough, recurrent infections, fever, weight loss, or loss of appetite. A Computed Tomography (CT) scan demonstrated a triangular mass in the medial left lower lobe measuring 2.7 × 3.2 × 3.6 cm abutting the pleura near the descending thoracic aorta (Fig. 1). A Positron Emission Tomography (PET) scan performed in March 2023 showed a non-FDG-avid mass with mild peripheral flurodeoxyglucose (FDG) activity, suggestive of adjacent atelectasis, raising the possibility of sequestration or a bronchogenic cyst rather than neoplasia.

**Fig. 2:**
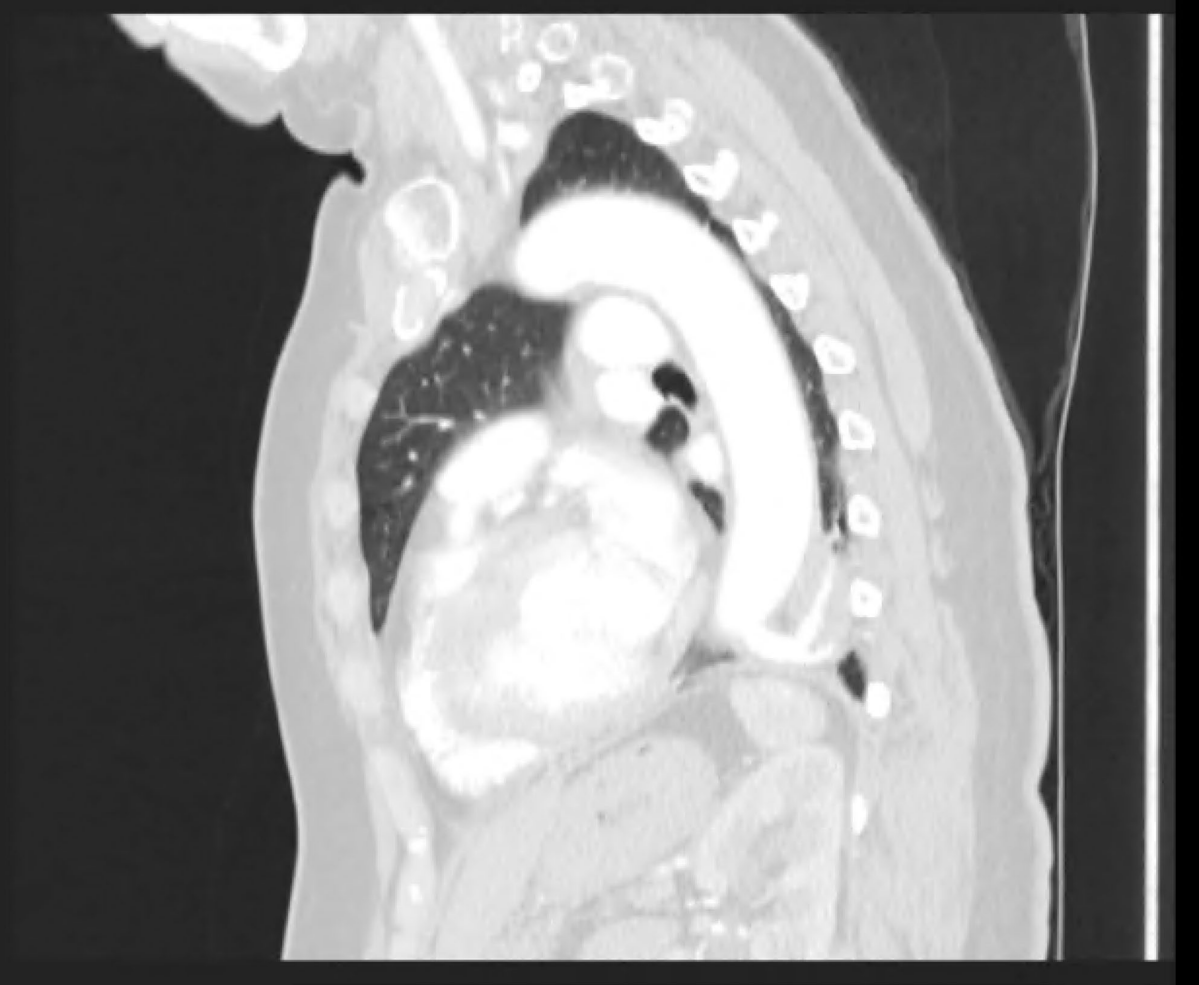
CT Chest with contrast sagittal view showing feeding vessel from descending thoracic aorta

A Computed Tomography Angiography (CTA) done in October 2024 revealed a feeding artery from the descending aorta consistent with pulmonary sequestration (Fig. 2). Follow-up CT in June 2025 showed a stable 3.1 cm mass with a feeding arterial supply, consistent with prior findings, without any suspicious pulmonary nodules. The patient remained asymptomatic and denied cough, hemoptysis, fever, or weight loss. She has a notable family history of malignancies, including lung cancer in a sibling, hepatic cancer in her mother, and lymphoma in her daughter. On examination, she had normal respiratory and cardiovascular findings, with no clubbing, cyanosis, or lymphadenopathy. Surgical intervention was offered, but the patient opted for routine observation and surveillance instead. She follows up with her pulmonologists once every 6 months, with imaging done during the office visit. The lesion appeared stable until the end of her 3-year follow-up, with plans for surgical resection in case the patient developed recurrent infections/hemoptysis or concerning change in the lesion size.

## Discussion

The etiology of sequestrations has been the subject of great debate. The most widely accepted theory, which provides a single mechanism for the spectrum of pathology described in the literature, suggests that PS result from the formation of an accessory lung bud caudal to the normal lung buds. The earliest description of PS was provided by Pryce et al. in 1946, who suggested that PS results from the persistence of systemic arteries that exert traction on the lung, causing a portion to separate from the main lung mass [3]. On the other hand, several authors have suggested that a systemic arterial supply may be recruited or develop in response to lung infection. Although popular in an era when most PS were presented because of infection, the increasing number detected antenatally suggests that this is highly unlikely to account for the majority. Furthermore, this theory does not easily account for Extralobar Sequestration (ELS) or Communicating Bronchopulmonary Foregut Malformation (CBPFM) [4].

### Incidence

Congenital pulmonary malformations have an estimated incidence of 2.2–6.6%, making them rare compared to acquired lung diseases. Notably, the quality of prenatal imaging is known to be adversely affected by several factors, likely leading to underdetection of anomalies [4]. The increase in case detection during adulthood is likely due to diagnostic advancements [5]. In a case series from Mayo Clinic, 22.7% of patients who had PS did not complain of any specific symptoms and were diagnosed incidentally on chest CT scans during health examination [6].

### Classification

The first description of an aberrant systemic artery supplying the lung was offered by Huber in 1777. The anomalous artery he observed arising from the thoracic aorta supplied the right lower lobe of the normal lung. Pryce’s clear description of intralobar sequestration (ILS) in 1946 established pulmonary sequestration as a distinct clinical entity [3, 7].

Two types of sequestration are described in the modern literature. An extra-lobar sequestration (25%) has its own pleura and drains into the systemic veins (azygos vein or vena cava), whereas an intra-lobar sequestration (75%) is included within the visceral pleura of the normal lung and drains into one of the pulmonary veins.

Of note, the extralobar form is more commonly associated with congenital developmental disorders. Approximately 10% of detected extralobar pulmonary sequestra are noted below the diaphragm. By contrast, the intralobar form is more common in adulthood, although most intralobar sequestrations are identified in patients under 20 years of age.

### Diagnosis

The diagnosis of ELS and ILS is based on imaging studies. ELS lesions generally receive their blood supply from the thoracic aorta and are more often detected by prenatal ultrasound, as their extrapulmonary location makes them easier to visualize. ELS is usually asymptomatic, though torsion and infarction of ELS have also been reported as the presenting feature [8].

ILS lesions are often not detected until adolescence or adulthood because they are hidden within normal lung parenchyma. Most are asymptomatic, and they are diagnosed incidentally by imaging. In addition to incidental computed tomography (CT) findings, the most common clinical presentation is recurrent infection [2].

The most common CT findings were mass lesions (50.5%) and cystic lesions (20.6%). Most lesions were located near the spine in the lower lobes. Arterial supply was mainly from the thoracic aorta (87.4%) and the abdominal aorta (10.5%). Intralobar and extralobar PS accounted for 90.7% and 9.3% of patients, respectively [6]. Most sequestrants have feeding vessels directly from the aorta. In contrast, some arise from smaller systemic arteries, such as the epigastric artery, left gastric artery, celiac trunk, pulmonary artery, or coronary artery [9–13]. Because of multiple anatomic variations, it is essential to obtain high-quality preoperative radiographic imaging to determine the most suitable surgical approach if surgical intervention is considered. Another essential tool for demonstrating anomalous arterial supply is Magnetic Resonance Angiography (MRA).

No tumor marker is currently available or studied for pulmonary sequestrants. However, Kong et al. described elevated Neuron-specific enolase (NSE) as a tumor biomarker at the time of diagnosis in their case report, which subsequently normalized after tissue resection [14]. This anecdotal report is an exception to the generalization, as no other case reports have identified NSE as a biomarker for pulmonary sequestrant.

### Complications

Chatelain et al. reported a patient with secondary dilated cardiomyopathy induced by a left-to-left shunt through the sequestration vessels. This shunt resulted in volume overload of the left ventricle. The patient’s condition significantly improved after surgical removal of the sequestrum [5]. The most reported complications of extralobar pulmonary sequestration in adults are pleural empyema, hemothorax, and hemoperitoneum. Intralobar pulmonary sequestration is often accompanied by recurrent unilateral pneumonia or hemoptysis. Notably, there has been reports of coexistent pulmonary sequestration and malignancies [5].

### Treatment

Although non-surgical embolization of the aberrant artery has been described, surgical resection remains the treatment of choice in operable patients.

Options include minimally invasive procedures such as video-assisted thoracoscopic surgery (VATS) and robotic-assisted thoracoscopic surgery (RATS) [6]. Open thoracotomy can be more invasive, associated with greater intraoperative blood loss, longer postoperative hospital stays, and longer duration of chest drainage [6]. Laura et al. described an anatomical variant requiring a hybrid approach of angiographic embolization followed by surgery [2].

Although surgical resection has traditionally been recommended for pulmonary sequestration, the decision to intervene is a clinical one made in accordance with the patient’s values and preferences. In the appropriate setting, observation with serial imaging may be considered [9], though surgery is usually recommended for large, cystic lesions, including in asymptomatic patients. Xiaoqian et al. reported a pseudoaneurysm of the feeding artery and its eventual rupture in a patient who was being followed with serial imaging [15]. Mohammad et al., in their retrospective study comparing resected and non-resected adult pulmonary sequestration, reported that patients without resection had no complications during their median follow-up of 19 months [16]. Surgical resection carries significant risk as well, with a mortality rate up to 12.5% [17]. Endovascular and hybrid techniques, offer lower risk of complications, but evidence is limited to case series, and larger controlled trials studying their benefits are still lacking [18]. Song et al., in their retrospective study, noted that 96% of unresected pulmonary sequestration patients who were initially symptomatic remained asymptomatic for a median follow-up of 21 months. They recommend that nonsurgical follow-up can be considered for asymptomatic patients, considering the indolent nature of the disease [19].

This patient’s lesion exhibited hallmark features of sequestration—arterial supply from the systemic circulation (descending aorta) and absence of FDG avidity on PET, which helps differentiate it from malignant neoplasms. Given the absence of symptoms such as recurrent pneumonia, hemoptysis, or significant compression, surgical resection was deferred. Instead, a conservative approach with serial imaging was adopted. Previous studies have reported that the mean age of diagnosis is 35–45 years, and PS diagnosed after age 65 is a clinical exception, though not clinically non-existent. Yong et al., in their retrospective analysis of adult and pediatric PS, noted that the maximal reported age of PS was 77 [20].

Our report highlights the importance of comprehensive evaluation using multimodality imaging, including CT, CTA, and PET/CT, to avoid unnecessary invasive procedures in asymptomatic patients. The management approach aligns with recommendations that asymptomatic pulmonary sequestrations without complications may be observed, reserving surgery for those with recurrent infections or suspicious growth. To our knowledge, very few cases have reported incidentally detected, asymptomatic intralobar sequestration diagnosed by the age of 70 and successfully managed non-operatively over several years.

## Conclusion

Pulmonary sequestration, though rare, should be included in the differential diagnosis of non-FDG-avid pulmonary masses with systemic arterial supply. In asymptomatic cases like this, close radiologic surveillance remains a reasonable management strategy.

## Data Availability

No datasets were generated or analysed during the current study.
